# Role of Peripheral Coordination Boron in Electrocatalytic Nitrogen Reduction over N-Doped Graphene-Supported Single-Atom Catalysts

**DOI:** 10.3390/molecules28124597

**Published:** 2023-06-07

**Authors:** Ruijie Ma, Xintong Weng, Linghui Lin, Jia Zhao, Fenfei Wei, Sen Lin

**Affiliations:** State Key Laboratory of Photocatalysis on Energy and Environment, College of Chemistry, Fuzhou University, Fuzhou 350002, China

**Keywords:** N-doped graphene, electrocatalytic nitrogen reduction, single-atom catalysts, coordination environment, density functional theory

## Abstract

In this work, we investigate the effect of peripheral B doping on the electrocatalytic nitrogen reduction reaction (NRR) performance of N-doped graphene-supported single-metal atoms using density functional theory (DFT) calculations. Our results showed that the peripheral coordination of B atoms could improve the stability of the single-atom catalysts (SACs) and weaken the binding of nitrogen to the central atom. Interestingly, it was found that there was a linear correlation between the change in the magnetic moment (μ) of single-metal atoms and the change in the limiting potential (U_L_) of the optimum NRR pathway before and after B doping. It was also found that the introduction of the B atom suppressed the hydrogen evolution reaction, thereby enhancing the NRR selectivity of the SACs. This work provides useful insights into the design of efficient SACs for electrocatalytic NRR.

## 1. Introduction

Ammonia (NH_3_) is an important chemical product for the chemical industry [1,2,3]. It is widely used in the production of fertilizers, plastics, synthetic fibers, and other chemicals and serves as a promising medium for hydrogen storage [4,5,6]. The Haber–Bosch process is the primary route for the industrial synthesis of ammonia. This method requires harsh reaction conditions (400–600 °C, 150–300 atm) and is accompanied by significant CO_2_ emissions during the reaction process [5,7,8,9]. To overcome these challenges, there is growing interest in the development of electrocatalysts that are recyclable and sustainable. The electrocatalytic nitrogen reduction reaction (NRR) is expected to achieve the direct conversion of nitrogen to ammonia in water at ambient temperature and pressure [10]. In the electrochemical system, protons and electrons are obtained by the electrolysis of water, and the only raw materials required for the ammonia synthesis process are water, nitrogen (from the air), and electrical energy. Under acidic conditions, the anodic reaction takes place—3H_2_O → 3/2O_2_ + 6H^+^ + 6e^−^—and the cathodic reaction takes place—N_2_ + 6H^+^ + 6e^−^ → 2NH_3_ [11,12,13]. 

Single-atom catalysts (SACs) [14] are widely used in electrocatalysis [15,16], thermal catalysis [17,18,19,20,21], and photocatalysis [22,23] due to their tunable coordination environment [24], high atomic utilization [25], high selectivity [26], and high activity [26]. The excellent catalytic performance of SACs can be achieved by selecting suitable metal centers [27]. Importantly, the catalytic performance of SACs can also be tuned by adjusting their coordination environment [24,28]. In recent years, nitrogen-carbon-material-supported transition metal SACs have been widely used in chemical reactions, such as the NRR, the oxygen evolution reaction (OER), the oxygen reduction reaction (ORR), water splitting, and the carbon dioxide reduction reaction (CO_2_RR) [23,29,30,31,32]. In particular, N-doped graphene-supported transition metal SACs (TMNx−Gs) have been widely used in various electrocatalytic reactions, both experimentally and theoretically [33,34]. For example, Ling et al. investigated the effect of varying the nitrogen coordination environment on the electrocatalytic NRR of TMNx−Gs using high-throughput calculations [35]. Wang et al. systematically studied the activity and selectivity of TMN_4_−Gs as a catalyst for the nitrate reduction reaction and explored the reaction mechanisms and the origin of the activity in detail. Zang et al. showed that Cu-N-C with four nitrogen atoms coordinated to a single-atom center had good NRR electrocatalytic activity [36].

Interestingly, Li et al. reported that the electronegativity of the coordination nitrogen atom resulted in an overpositive charge on the central atom of FeN_4_−G, which hindered the adsorption of intermediates and affected the catalytic activity of the NRR [37]. Furthermore, Li et al. found that the spin magnetic moment of FeN_4_-graphene was divided into two segments due to its high symmetry, which was detrimental to the nitrogen reduction reaction [38]. Recent studies have shown that the symmetry of N-doped graphene-supported metal SACs (denoted as TMN_4_−Gs) is broken after doping the peripheral coordination environment with foreign elements, such as S, P, and B [3,39,40]. In this case, the spin state of the metal site is altered, resulting in enhanced electrocatalytic activity. Similarly, Zhang et al. found that the electrocatalytic activity of the OER and ORR could be enhanced by doping B atoms near the N-doping site of MnN_4_-Gs [39]. By combining density functional theory (DFT) and machine-learning (ML) methods, Shu et al. demonstrated that the number of unpaired d electrons in transition metals played a key role in the NRR process [41] because the number of unpaired d electrons directly affected the spin magnetic moment [42]. Therefore, it can be speculated that, when a B atom is introduced into the peripheral coordination sphere of a TMN_4_−G, it can create an asymmetric coordination geometry and induce a redistribution of the electron density, thus triggering the spin polarization of the TMs. This offers the possibility of achieving good electrocatalytic NRR activity.

In the current work, the catalytic NRR of TMN_4_-G and B-doped TMN_4_-G (denoted as TMN_4_B−G) SACs is systematically investigated through DFT calculations. The results show that the stability of the SACs is improved after doping with B, but the overall nitrogen adsorption is weakened. Nine TMN_4_−G (TM = Sc, Ti, V, Cr, Zr, Nb, Mo, Ru, and Hf) SACs are screened for stability and N_2_ adsorption ability, followed by comparison of their electrocatalytic activity and selectivity before and after B doping. The different SACs show different changes in catalytic activity and selectivity after doping with B atoms, with Mo SACs showing better NRR activity than the other SACs and good NRR selectivity, which can be attributed to the fact that doping of the peripheral coordination B atoms leads to spin polarization of the active site. In addition, we find that there is a linear correlation between the change in the magnetic moment (μ) of metal atoms before and after doping and a change in the limiting potential (U_L_) of the optimal NRR pathway. This work provides insight into the design of high-performance NRR electrocatalysts.

## 2. Computational Details

All spin-polarized DFT calculations were performed in the Vienna Ab-initio Simulation Package (VASP) with the gradient-corrected Perdew–Burke–Ernzerhof (PBE) functional [43,44,45]. A plane-wave basis set was used for the valence electrons, and the energy cutoff was set to 400 eV. The core electrons were described using the projector-augmented wave (PAW) method [46,47]. The graphene model consisted of a 7 × 7 × 1 supercell and a 15 Å vacuum layer along the z-axis to avoid the interaction between two adjacent periodic images. A Monkhorst−Pack mesh of 2 × 2 × 1 was used for geometry optimization. During the geometry optimization process, all atoms were fully relaxed, and the convergence criteria for energy and force were 10^−4^ eV and 0.02 eV/Å, respectively.

The Gibbs free energy change (ΔG) in each hydrogenation step was calculated using the computational hydrogen electrode (CHE) model proposed by Nørskov et al. [48,49]. On this basis, the ΔG of the NRR process was calculated using the following equation:

(1) $$\Delta G=\Delta E+{\Delta E}_{zpe}-T\Delta S+{\Delta G}_{pH}+eU,$$

where ΔE is the electronic energy difference between the reactant and the product on the catalyst surface in the NRR process, which can be calculated directly from DFT calculations; ΔE_zpe_ is the zero-point energy correction, which can be obtained from the vibration frequency calculation (Table S1, Supporting Information); T is the temperature (T = 300 K); and ΔS is the entropy change, which can be obtained from the NIST database. ΔG_pH_ is the pH free energy correction, and its calculation formula is given by the following:

(2) $$\Delta G_{\mathrm{pH}}=\ln10{\;\times \;k}_{B}T\;\times \;\mathrm{pH},$$

where k_B_ is the Boltzmann constant, and the value of pH in this work is zero. The e and U in the calculation formula represent the number of electrons transferred and the applied electrode potential, respectively. In addition to this, we chose a limiting potential to describe the catalytic activity indicator in the NRR, which can be derived from the free energy change in the potential-determining step:

(3) $$U_{L}=-{\Delta G}_{\max}/e,$$

where ΔG_max_ is also the Gibbs free energy variation in the potential-determining step (ΔG_PDS_) [50]. 

The stability of the catalyst was evaluated by its binding energy (E_b_) and cohesive energy (E_c_). The binding energy of the TM supported on N-doped graphene was obtained by the following:

(4) $$E_{b}=E_{{\mathrm{TMN}}_{4}(B)-G}-E_{N_{4}(B)-G}-E_{\mathrm{TM}-\mathrm{single}},$$

where E_TMN_4_(B)-G_ is the total energy of the TMN_4_(B)-G catalysts, E_N_4_(B)-G_ is the energy of the N(B)-doped graphene, and E_TM-single_ is the energy of a single transition metal atom. The stability of a metal atom in the bulk metal can be described by the cohesive energy:

(5) $$E_{c}=E_{\mathrm{TM}-\mathrm{bulk}}/n-E_{\mathrm{TM}-\mathrm{single}},$$

where E_TM-bulk_ is the energy of the transition metal bulk, and n is the number of transition metal atoms in the bulk. The value (E_b_ − E_c_) is usually used as a measure of whether a catalyst is agglomerated. Negative values indicate that single atoms do not readily agglomerate into metal clusters [51,52]. 

## 3. Results and Discussion

**Figure 1:**
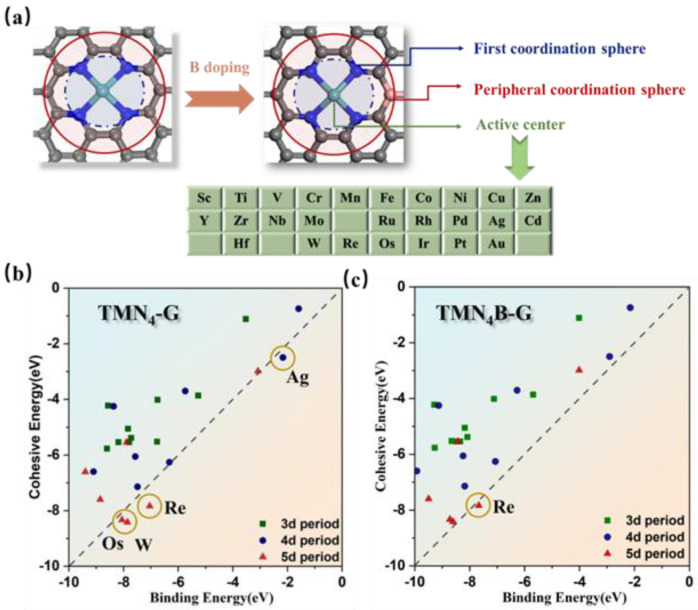
(**a**) The coordination environment of an N-doped graphene-supported single-atom catalyst before and after B doping. The cyan, blue, grey, and pink balls represent the transition metal, nitrogen, carbon, and boron atoms, respectively. The green table shows the 26 transition metals employed. Schematic diagrams comparing the binding and cohesive energies of TMN_4_−G (**b**) and TMN_4_B−G catalysts (**c**). The dashed line indicates that *E*_b_ is equal to *E*_c_. Above the dashed line indicates that *E*_b_ − *E*_c_ < 0, i.e., the SACs are stable.

In this work, we chose twenty-six transition metal atoms to be supported on N-doped graphene, as shown in Figure 1a. In the first coordination sphere, four N atoms were bonded to the TM as the active center. To investigate the role of the B atoms in the peripheral coordination spheres for electrocatalytic NRR, we also constructed B-doped catalysts. Previous studies have shown that the following conditions need to be fulfilled for the design of efficient single-atom catalysts for NRR: (1) single transition metal atoms do not form nanoparticles on a support; (2) N_2_ should have good adsorption on the catalyst surface; (3) the catalyst should be able to activate N_2_ efficiently with good NRR reactivity; and (4) the NRR selectivity should be higher than the hydrogen evolution reaction (HER) [51,53,54]. Next, the NRR catalytic performances of the SACs are evaluated based on these four criteria.

### 3.1. Stability of TMN_4_B−G and TMN_4_−G Catalysts

The stability of catalysts is a fundamental prerequisite for achieving high-performance catalysis. Here, we screened the stability of the catalysts by comparing the binding and cohesive energies. The binding and cohesive energy data for the TMN_4_−G catalysts are shown in Figure 1b. It can be found that most of the SACs were stable, with the exception of the SACs for Ag, Re, Os, and W. Figure 1c compares the binding and cohesive energy data for TMN_4_B-G catalysts, showing that the doping of B atoms improved the stability of the catalysts, but the Re SAC remained unstable. The surface structures of TMN_4_−G and TMN_4_B−G catalysts after the optimization process are shown in Figures S1 and S2, respectively. We can clearly see that the difference between the highest and lowest atoms along the z-axis in the structure of the TMN_4_−G catalysts was between 0.03 Å and 1.39 Å. In contrast, the doping with B atoms resulted in a pronounced corrugation on the catalyst surface, with the difference between the highest and lowest atoms along the z-axis being even larger, between 0.49 Å and 1.94 Å. Thus, the presence of surface corrugations could help to weaken the electron repulsion of the nitrogen lone pair and, thus, improve the stability of the catalyst [31,55,56]. We further analyzed the origin of the enhanced stability with B doping through the electronic structure. Figure S3 shows the partial density of states (PDOS) of Ag (4d), W (5d), Os (5d), and the four coordination N (2p) for TMN_4_−G (a,c,e) and TMN_4_B−G (b,d,f) catalysts. The results showed a strong peak near the Fermi level after B doping. This means that the interaction between W and the adjacent N atoms was strengthened, i.e., the SAC became more stable. This may be caused by the d orbitals of the metal and the p orbitals of N moving closer to the Fermi energy level due to the electron deficiency of B. In the following, we select only the stable SACs to investigate the effect of the peripheral coordination of the B atoms on the electrocatalytic NRR.

### 3.2. N_2_ Adsorption on TMN_4_B−G and TMN_4_−G Catalysts

**Figure 2:**
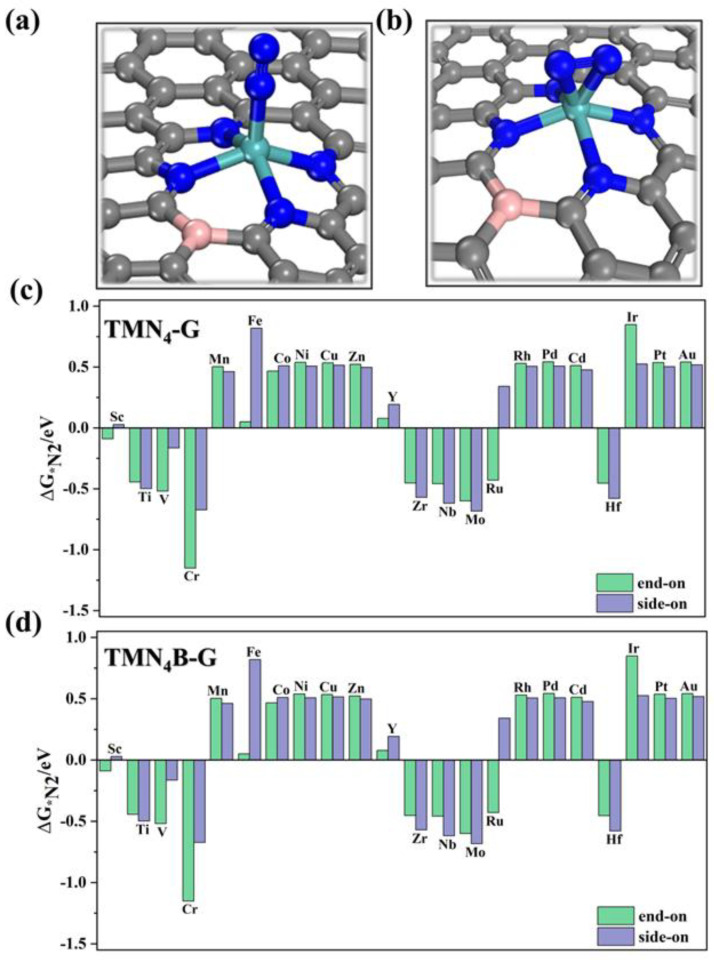
The structures of (**a**) end−on and (**b**) side−on adsorption configurations for N_2_. The cyan, blue, grey, and pink balls represent the transition metal, nitrogen, carbon, and boron atoms, respectively. (**b**) Schematic diagrams of the adsorption free energy (ΔG_*N2_) of N_2_ on TMN_4_−G (**c**) and TMN_4_B−G (**d**) catalysts for the end-on and side-on adsorption patterns.

The adsorption of N_2_ on a catalyst surface is a key step in the NRR. There are two configurations of N_2_ adsorption on the active site. One is the “end-on” adsorption configuration, in which one N atom is bound to a transition metal atom, and the other N is warped, as shown Figure 2a. The other is a “side−on” adsorption configuration, where two N atoms are bound to the transition metal atoms, as shown Figure 2b. Figure 2c,d show the change in Gibbs free energy (∆G_*N2_) of N_2_ adsorption on the surface of the TMN_4_B−G catalysts. It can be found that the adsorption of N_2_ by the SACs of the late transition metal elements was significantly weaker than that of the early ones. It is generally accepted that the metal atom provides an empty d orbital to accept the lone pair of electrons from the N_2_ while providing electronic feedback to the N_2_, thereby enhancing the interaction between the metal atom and the N_2_ species [35,57,58,59]. As a result, TMN_4_B−G SACs with late transition metals were less able to adsorb N_2_ due to the lack of empty d orbitals in the late transition metals.

By comparing the results in Figure 2c,d, it can be seen that the adsorption of N_2_ by SACs was weakened after B doping (except for Nb). Among these, we took the Cr SAC as an example to analyze its electronic structure since it showed the largest change in N_2_ adsorption. From the charge density difference and Bader charge transfer calculations shown in Figure S4a,b, it is clear that, after B doping, fewer electrons were fed back to N_2_ from the central atom, which weakened the interaction between the central atom and N_2_. Structurally, the distance between the TM and N in nitrogen was elongated. In addition, the bond length of N≡N became shorter, and the activation of N≡N was reduced after B atom doping. Figure S4c shows that the comparison of the PDOS of Cr(3d) and *N_2_(2p) for the Cr catalysts before and after B doping also supported this view. Compared to CrN_4_B-G, CrN_4_-G had a strong peak near the Fermi level, indicating a stronger interaction of the central atom with the adsorbed nitrogen and a stronger adsorption of N_2_.

Previous studies have shown that, when ΔG_*N2_ is greater than 0 eV, the adsorption is weak, and it is difficult to proceed with the subsequent hydrogenation reaction [35,57,58,59]. An analysis of the free energy change in N_2_ adsorption of the SACs in Figure 2 showed that there were nine metals with a ΔG_*N2_ less than 0 eV, namely Sc, Ti, V, Cr, Zr, Nb, Mo, Ru, and Hf. In order to better explore the effect of B doping, we next also investigate the NRR performances of the above nine single-atom catalysts.

### 3.3. Performance of NRR

**Figure 3:**
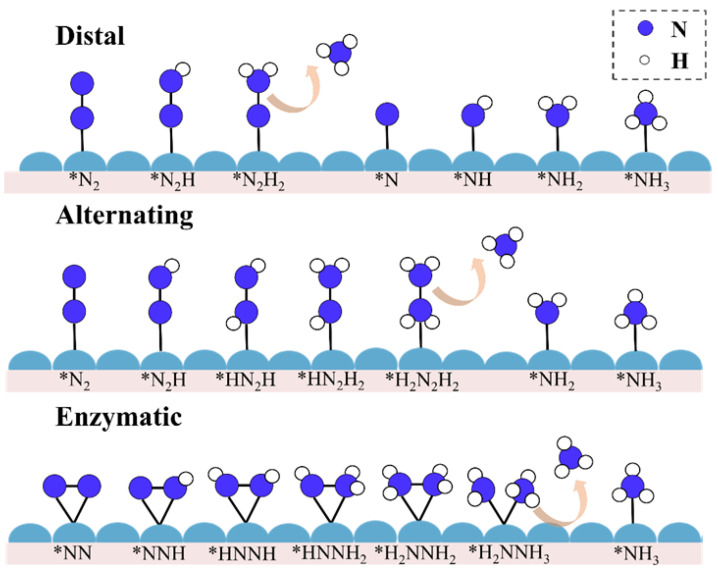
Schematic representation of three possible pathways for the NRR on the surface of an electrocatalyst. The dark blue balls represent nitrogen atoms, the white balls represent hydrogen atoms, and the light blue bumps represent the active site. The * denotes the adsorption position.

According to the two configurations of nitrogen adsorption, there are three reaction pathways for the NRR, namely the distal, alternating, and enzymatic mechanisms (Figure 3). When N_2_ is adsorbed in the end-on mode, there are two types of hydrogenation patterns, namely the distal and alternating mechanisms. For the distal mechanism, the first three hydrogenation processes occur on the distal N atom until the first ammonia molecule is formed and desorbed. The last three hydrogenations then take place on the remaining N atom to form a second ammonia molecule. The alternating mechanism works as follows: six H^+^/e^−^ cycles alternate between the two N atoms, and two NH_3_ molecules are formed in sequence. When N_2_ is adsorbed following the side-on adsorption mode, the hydrogenation process alternates between the two N atoms, and two ammonia molecules are formed in turn. This reaction pathway is known as the enzymatic mechanism.

**Figure 4:**
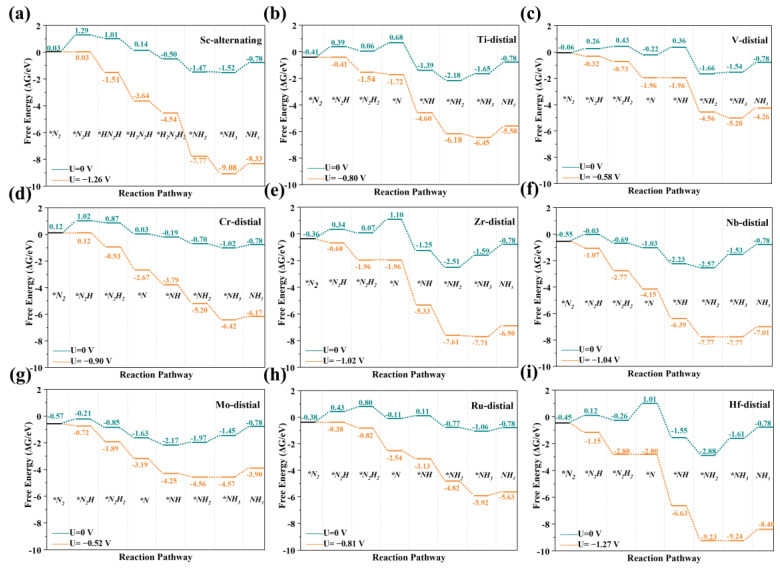
The optimal Gibbs free energy paths of the NRR of ScN_4_B−G (**a**), TiN_4_B−G (**b**), VN_4_B−G (**c**), CrN_4_B−G (**d**), ZrN_4_B−G (**e**), NbN_4_B−G (**f**), MoN_4_B−G (**g**), RuN_4_B−G (**h**), and HfN_4_B−G (**i**), respectively.

**Figure 5:**
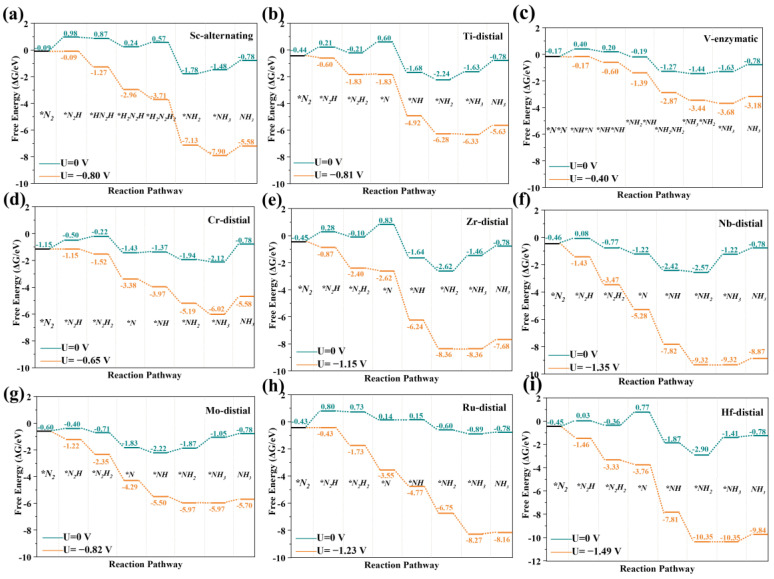
The optimal Gibbs free energy path of the NRR of ScN_4_−G (**a**), TiN_4_−G (**b**), VN_4_−G (**c**), CrN_4_−G (**d**), ZrN_4_−G (**e**), NbN_4_−G (**f**), MoN_4_−G (**g**), RuN_4_−G (**h**), and HfN_4_−G (**i**), respectively.

To assess the NRR activity of the SACs, we calculated the free energy changes in the NRR for all the TMN_4_−G and TMN_4_B−G catalysts according to the above three mechanisms. It should be noted that CrN_4_−G and RuN_4_(B)−G had no side-on adsorption after structural optimization, and the free energy change in N_2_ absorbed on the ScN_4_(B) −G surface following the side-on adsorption mode was greater than 0 eV. Therefore, for CrN_4_-G, RuN_4_(B) −G, and ScN_4_(B) −G, only the distal and alternating pathways were considered. In all the elementary steps, the U_L_ was the minimum potential required to overcome the energy barrier of the potential-determining step. The path with the minimum U_L_ was defined as the optimal one. Taking MoN_4_B−G and MoN_4_−G as examples, the optimized adsorption configurations for the intermediates are shown in Figure S5. The optimal Gibbs free energy paths for the TMN_4_B−G (TM = Sc, Ti, V, Cr, Zr, Nb, Mo, Ru, and Hf) and TMN_4_−G (TM = Sc, Ti, V, Cr, Zr, Nb, Mo, Ru, and Hf) catalysts are shown in Figure 4 and Figure 5, respectively. In addition, the free energies of other pathways at U = 0 V for the TMN_4_−G and TMN_4_B−G catalysts are listed in Tables S3–S11, where the numbers in italics represent the ΔG_PDS_. The ΔG_PDS_ values of TiN_4_B-G, VN_4_B−G, CrN_4_B−G, MoN_4_B−G, and RuN_4_B−G were 0.80 eV, 0.58 eV, 0.90 eV, 0.52 eV, and 0.81 eV, respectively. Compared to Ru(0001) (ΔG_PDS_ = 0.98 eV), which has excellent NRR catalytic activity [60,61], the above catalysts could be considered as promising catalysts. The NRR catalytic performances of Zr SAC, Nb SAC, and Hf SAC were lower with or without the introduction of B atoms. ScN_4_−G, TiN_4_−G, VN_4_−G, CrN_4_−G, and MoN_4_−G showed better activity after B doping. In contrast, RuN_4_-G, which originally had poor NRR catalytic activity, showed good NRR catalytic activity after the introduction of the B atom. Interestingly, doping with B atoms changed the potential-determining step of the NRR. Among them, the Ti SAC changed from the third step of the hydrogenation reaction to the first step of the hydrogenation reaction, the V SAC changed from the first step of the hydrogenation reaction to the fourth step, and both the Zr and Hf SACs change from the last step of the hydrogenation reaction to the third step. In this case, both the mechanism of the NRR process and the electrocatalytic NRR activity of the SACs were changed. 

**Figure 6:**
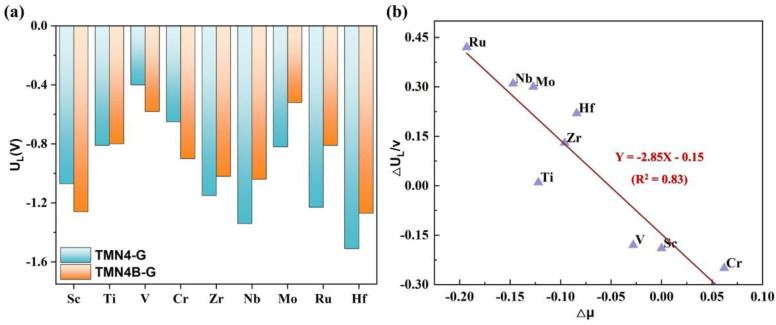
(**a**) The limiting potential of TNMN_4_−G and TMN_4_B−G catalysts with different metals at the optimal path. (**b**) The relationship between the change in the spin magnetic moment and the change in the limiting potential before and after B doping.

As shown in Figure 6a, by comparing the U_L_ of the best reaction pathways of the TMN_4_B−G and TMN_4_−G catalysts, we found that the doping of peripheral B atoms significantly enhanced the electrocatalytic NRR activities of Ti, Zr, Nb, Mo, Ru, and Hf, and the U_L_ of their optimal pathways increased by 0.01 V, 0.13 V, 0.31 V, 0.30 V, 0.42 V, and 0.24 V, respectively. However, the electrocatalytic NRR activities of Sc, V, and Cr became lower after doping with B atoms because the U_L_ of their optimal pathways decreased by 0.19 V, 0.09 V, and 0.25 V, respectively.

Next, we explored the effect of B doping on the magnetic moment of the central single atom. Previous studies have suggested that the activity of NRR SACs may be related to the unpaired d electrons of supported single atoms [41] and that there is a correlation between the number of unpaired electrons (n) and μ:

(6) $$\mu =\sqrt{n\left(n+2\right)}\;\mu_{B}$$

Therefore, we further investigated the relationship between the value of the change in the magnetic moment (Δμ = μ_TMN4B-G_ − μ_TNMN4-G_) and the value of the change in the limiting potential of the optimal path (ΔU_L_ = U_L-TMN4B-G_ − U_L-TMN4-G_), as shown in Figure 6b. If ΔU_L_ < 0, it meant that the U_L_ of the catalyst was greater after doping with B atoms, i.e., the performance of the catalyst was better. This relationship is shown in Figure 6b, with the R^2^ equal to 0.83. It can be seen that the ΔU_L_ increased as the Δμ decreased, indicating that the greater the μ of the central metal atom was reduced after B doping, the more positive the required limiting potential and the better the catalytic performance of the NRR. This finding provides insight to the design of better-performing NRR electrocatalysts by modulating the peripheral ligand environment.

### 3.4. Selectivity

**Figure 7:**
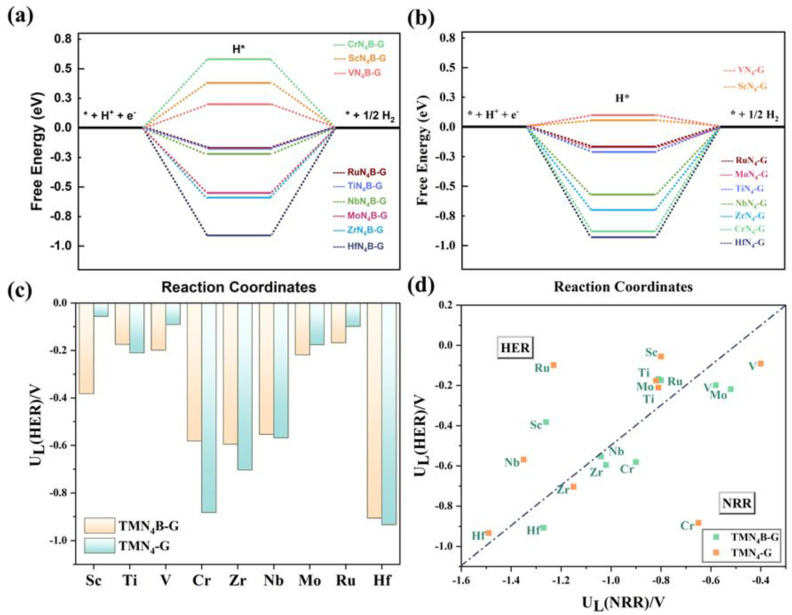
The Gibbs free energy for the HER of TMN_4_B−G (**a**) and TMN_4_−G (**b**) catalysts with different metals. The * denotes the adsorption position (**c**) The U_L_ of TNMN_4_−G and TMN_4_B−G catalysts with different metals for the HER. (**d**) Comparison of the limiting potential (U_L_) between the HER and NRR. Below the dotted line is U_L_(HER) − U_L_(NRR) < −0.5 V, and catalysts in this region were more inclined toward NRR. Above the dotted line is U_L_(HER) − U_L_(NRR) > −0.5 V, and the catalysts in this region tended toward HER.

It is well known that the HER is the main competitive reaction for the NRR. A good NRR catalyst should not only have good NRR activity, but also high NRR selectivity, i.e., the HER reaction should be suppressed [59,62]. We calculated the Gibbs free energy diagrams of the HER for the SACs before and after doping with B atoms, as shown in Figure 7a,b, respectively. It can be seen from Figure 7c that ScN_4_−G, VN_4_−G, MoN_4_−G, and RuN_4_−G had more negative U_L_ values for the HER after the introduction of B atoms, indicating that the HER process was suppressed. Before B doping, the HER proceeded more easily due to the moderate binding strength of hydrogen to the central atom on the surface of the TMN_4_-G catalysts (ScN_4_−G: ΔG_*H_ = 0.06 eV, VN_4_−G: ΔG_*H_ = 0.09 eV, MoN_4_−G: ΔG_*H_ = −0.17 eV, and RuN_4_−G: ΔG_*H_ = −0.10 eV), which was more favorable for hydrogen adsorption and desorption. However, with B doping, hydrogen species became difficult to adsorb or desorb on the surface of the TMN_4_B-G catalysts (ScN_4_B−G: ΔG_*H_ = 0.38 eV, VN_4_B−G: ΔG_*H_ = 0.19 eV, MoN_4_B-G: ΔG_*H_ = −0.21 eV, and RuN_4_B-G: ΔG_*H_ = −0.17 eV), resulting in the suppression of the HER. Finally, we evaluated the selectivity of the catalysts by comparing the difference between the U_L_(HER) and U_L_(NRR), i.e., if the difference between the U_L_(NRR) and U_L_(HER) of a catalyst was greater than −0.5 V (the benchmark for metallic catalysts) [63,64], the catalyst was more susceptible to the NRR. As shown in Figure 7d, SACs above the dashed line were more prone to HERs, while SACs below the dashed line had higher NRR selectivity. It can be seen that the introduction of B atoms generally increased the selectivity of the SACs. In particular, MoN_4_−G, NbN_4_−G, and HfN_4_-G tended to shift from HER to NRR after doping with B atoms.

## 4. Conclusions

In conclusion, we used DFT calculations to investigate the stability of N−doped graphene−supported SACs, as well as their adsorption of N_2_, NRR activity, and selectivity before and after doping of the peripheral B atoms. The introduction of B atoms was found to break the symmetry of the single-atom coordination on N-doped graphene, causing spin polarization of the central metal atom. The doping with B atoms enhanced the stability of the SACs and affected the reactivity and selectivity of the NRR. Importantly, a linear relationship was found between the change in the magnetic moment of the single-metal atoms and the change in the limiting potential of the optimum pathways before and after B doping, that is, the more the magnetic moment of the central metal atom was reduced after B doping, the more positive limiting potential required and the better the catalytic performance of the NRR. Furthermore, it was found that the introduction of B atoms could suppress the competitive HER process, thus improving the NRR selectivity of the SACs. Among the catalysts, MoN_4_B−G had high NRR catalytic activity due to a calculated low ΔG_PDS_ of 0.52 eV. Meanwhile, it had NRR selectivity, as the NRR was superior to the HER when doped with B atoms. This provides new insights into the design of SACs for the NRR.

## Data Availability

Data can be found in the manuscript.

## Supplementary Materials

The following supporting information can be downloaded at: https://www.mdpi.com/article/10.3390/molecules28124597/s1, Figure S1. The structures on the surface after the optimization process of TMN_4_B-G. The blue, grey, pink, and other colors of balls represent the nitrogen, carbon, boron, and transition metals, respectively. Figure S2. The structures on the surface after the optimization process of TMN_4_B-G. The blue, grey, and other colors of balls represent the nitrogen, carbon, and transition metals, respectively. Figure S3. The partial density of states (PDOS) of Ag (4d) and four N (2p) in coordination for SACs before B doping (a) and after B doping (b), respectively, and the PDOS of X (5d) (X = W, Os) and four N (2p) atoms in coordination for SACs before B doping (c,e) and after B doping (d,f), respectively. Figure S4. The charge differential density diagrams after the N_2_ adsorption of CrN_4_-G (a) and CrN_4_B-G (b). The iso-surface levels were 0.0025 e Å^−3^, and charge accumulation and consumption are shown in yellow and cyan, respectively. The cyan, blue, grey, white, and pink balls represent transition metal, nitrogen, carbon, hydrogen, and boron atoms, respectively. (c) The PDOS of Cr (3d) and *N_2_ (2p) of SACs before B doping and after B doping. Figure S5. Optimizations of intermediate adsorption configurations of MoN_4_-G (a) and MoN_4_B-G (b), respectively. Table S1. This table lists the zero-point energies of different adsorbed species. Note that *N_2_ represents the end-on adsorption configuration and *N*N represents the side-on adsorption configuration, where the * denotes the adsorption position. Table S2. The central atomic charge of each catalyst and the charge change after B atom doping. Tables S3–S11. The Gibbs free energy of non-optimal paths at U = 0 V for ScN_4_(B) −G (a), TiN_4_(B) −G (b), VN_4_(B) −G (c), CrN_4_(B) −G (d), ZrN_4_(B) −G (e), NbN_4_(B) −G (f), MoN_4_(B) −G (g), RuN_4_(B) −G (h), and HfN_4_(B) −G (i), respectively. The ΔG_PDS_ is in italics.

## Sample Availability

Samples of the compounds are available from the authors.
