# Supplementary material for: Role of Peripheral Coordination Boron in Electrocatalytic Nitrogen Reduction over N-Doped Graphene-Supported Single-Atom Catalysts
Source: Molecules. 2023 Jun 7;28(12):4597. doi: 10.3390/molecules28124597 (PMC10304113; doi:10.3390/molecules28124597)
Supplement: Supplementary file 1 [file molecules-28-04597-s001.zip › molecules-2427675-supplementary.pdf]

Supporting information

Role of peripheral coordination boron in electrocatalytic nitrogen reduction over N-doped graphene-supported single-atom catalysts

Ruijie Ma, Xintong Weng, Linghui Lin, Jia Zhao, Fenfei Wei, and Sen Lin*

State Key Laboratory of Photocatalysis on Energy and Environment, College of Chemistry, Fuzhou University, Fuzhou 350002, China

* Corresponding author: slin@fzu.edu.cn

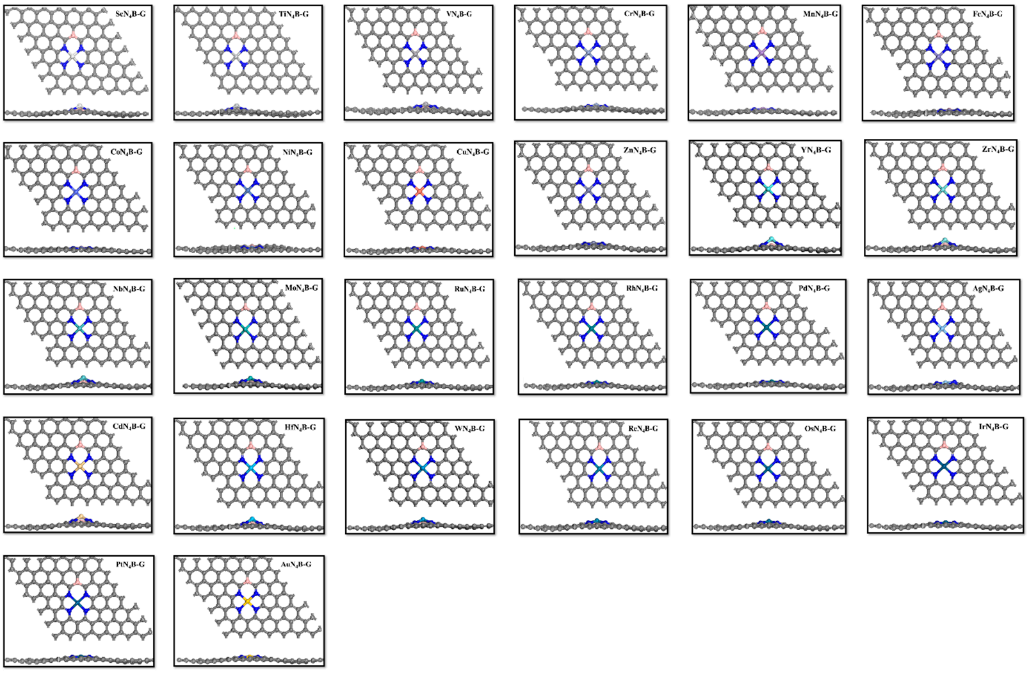

Figure S1. The structures of TMN_4B-G after optimization. The blue, grey, pink and other color balls represent nitrogen, carbon, boron and transition metals, respectively.

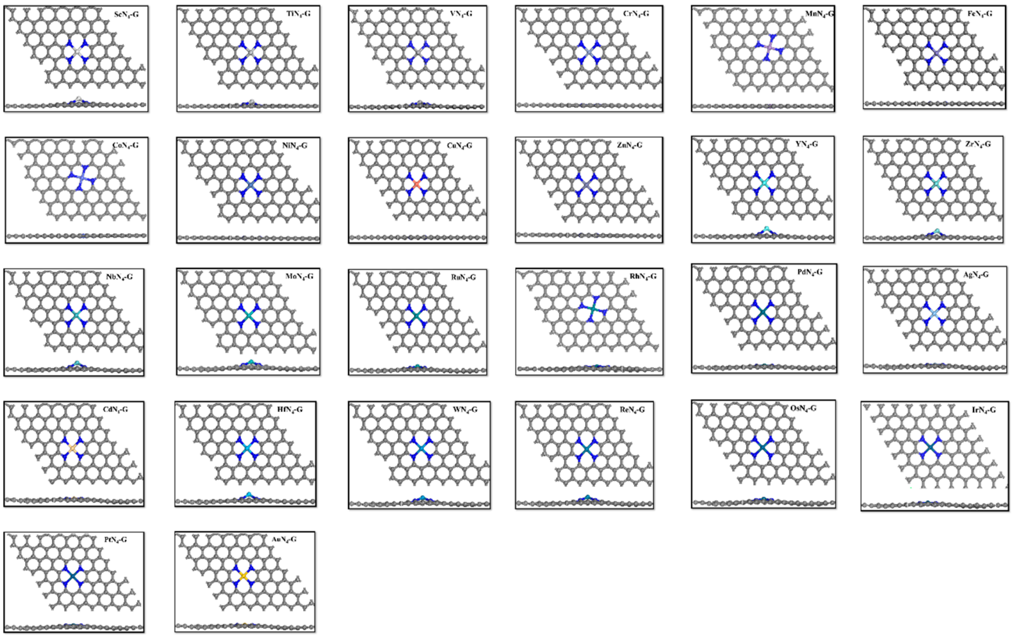

Figure S2. The structures of TMN_4-G after optimization. The blue, grey and other color balls represent nitrogen, carbon and transition metals, respectively.

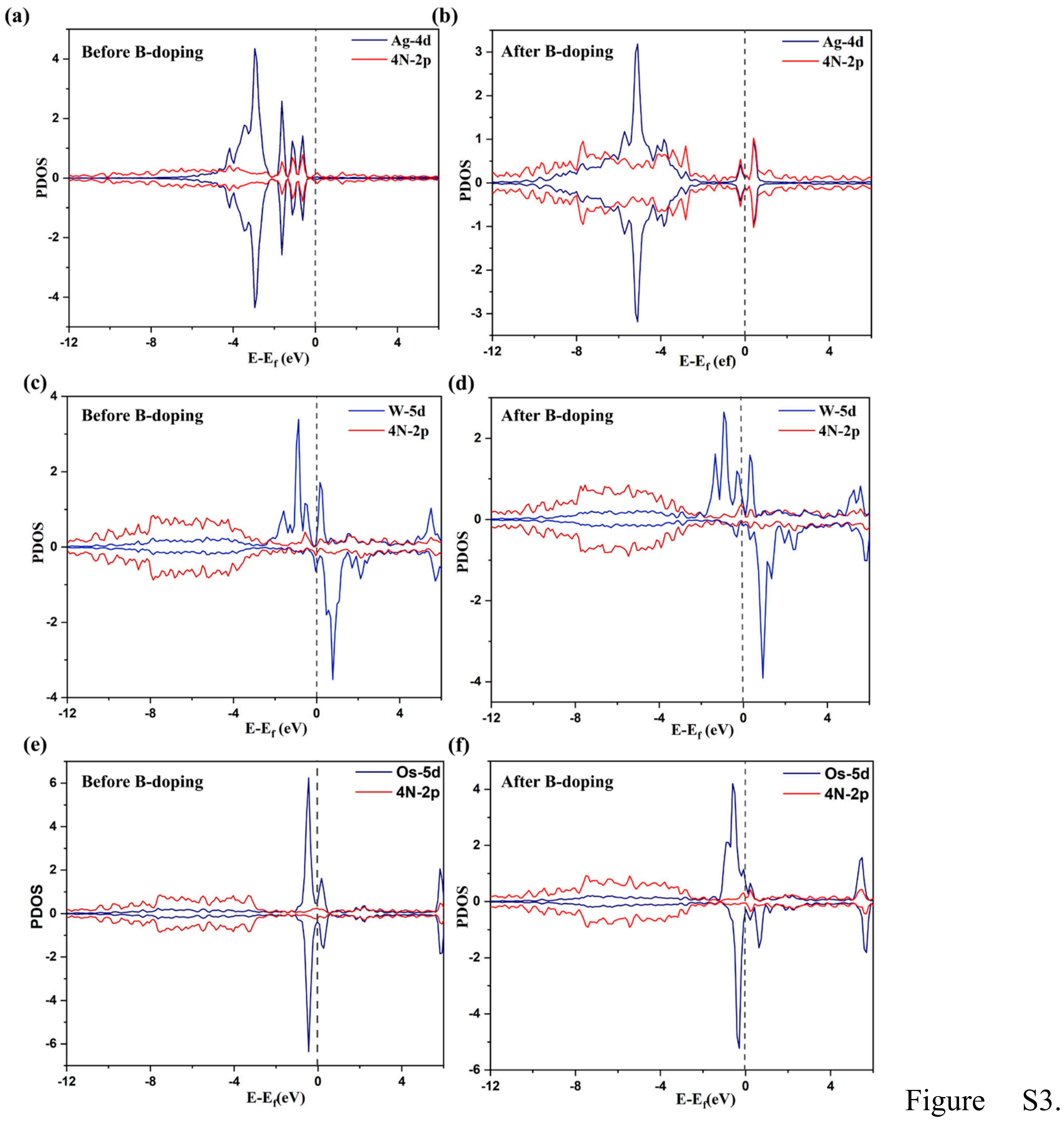

Figure S3. Partial density of states (PDOS) of Ag (4d) and four N (2p) atoms in the coordination for SAC before B-doping (a) and after B-doping(b), respectively. And PDOS of X (5d) (X= W, Os) and four N (2p) atoms in the coordination for SAC before (c, e) and after B-doping (d, f).

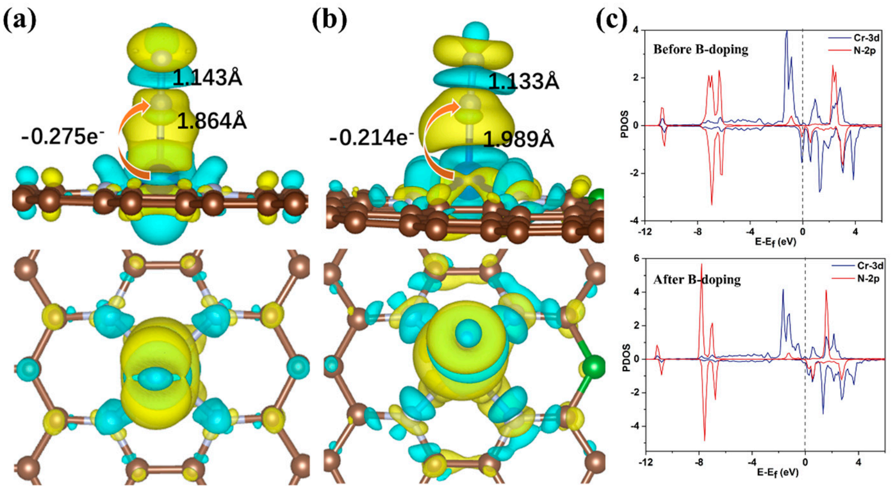

Figure S4. Charge differential density diagrams after N_2 adsorption on CrN_4-G (a) and CrN_4B-G (b). The iso-surface levels are 0.0025 e Å^{-3}, and the charge accumulation and consumption are shown in yellow and cyan, respectively. (c) PDOS of Cr (3d) and *N_2 (2p) for SAC before and after B-doping.

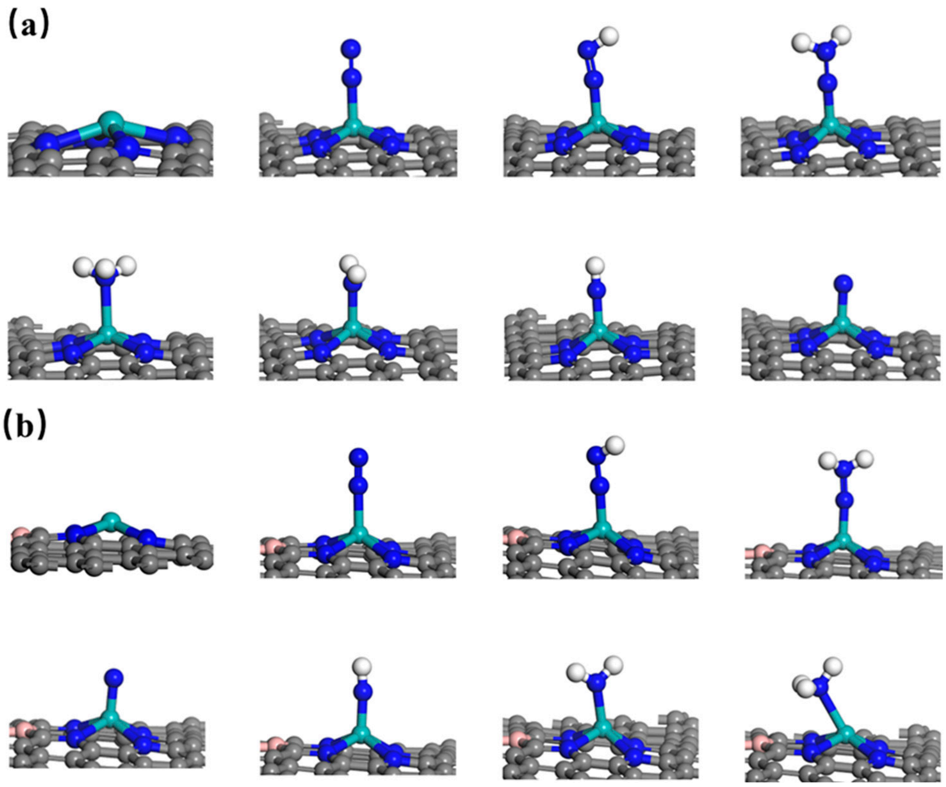

Figure S5. Optimized adsorption configuration of intermediates on MoN_4-G (a) and MoN_4B-G (b). The cyan, blue, grey, white and pink balls represent transition metal, nitrogen, carbon, hydrogen and boron atoms, respectively.

Table S1. The zero point energies of different adsorbed species. Note that $*N_2$ represents the end-on adsorption configuration and *N*N represents the side-on adsorption configuration, where the * denotes the adsorption position.

Species	E_{ZPE}(eV)
H	0.14
N_2	0.15
NH_3	0.89
$*N_2$	0.19
$*N_2H$	0.46
$*N_2H_2$	0.79
*N	0.08
*NH	0.34
$*NH_2$	0.63
$*NH_3$	1.01
*NHNH	0.83
$*NHNH_2$	1.15
$*NH_2NH_2$	1.49
*N*N	0.18
*N*NH	0.48
*NH*NH	0.79
$*NH*NH_2$	1.16
$*NH_2*NH_2$	1.47
$*NH_2*NH_3$	1.69

Table S2. The central atomic charge of each catalyst and the charge change after B atom doping.

TM	The Bader charge of TMN_4-G	The Bader charge of TMN_4B-G	Charge change
Sc	9.163	9.162	-0.0014
Ti	2.592	2.508	-0.084
V	3.675	3.626	-0.049
Cr	4.728	4.734	0.006
Zr	2.422	2.330	-0.092
Nb	11.431	11.366	-0.065
Mo	4.719	4.684	-0.035
Ru	7.096	7.124	0.028
Hf	2.407	2.310	-0.097

Table S3. The Gibbs free energy for distal pathway of ScN_4-G and ScN_4B-G at U=0 V. The ΔG_{PDS} is in italics.

Elementary step (distal)	Intermediate species	$\Delta G(ScN_4\text{-}G)$ /eV	$\Delta G(ScN_4B\text{-}G)$ /eV
$* + N_2 = *N_2$	$*N_2$	-0.09	0.04
$*N_2+(H^++e^-) =*NNH$	*NNH	1.13	1.32
$*NNH+(H^++e^-) =*NNH_2$	$*NNH_2$	-0.09	-0.11
$*NNH_2+(H^++e^-) =*N+NH_3$	*N	*1.57*	*1.57*
$*N+(H^++e^-) =*NH$	*NH	-2.07	-2.10
$*NH+(H^++e^-) =*NH_2$	$*NH_2$	-2.23	-2.19
$*NH_2+(H^++e^-) =*NH_3$	$*NH_3$	0.30	-0.051
$*NH_3=*+NH_3$	NH_3	0.70	0.75

Table S4. The Gibbs free energy for alternating and enzymatic pathway of TiN_4-G and TiN_4B-G at U=0 V. The ΔG_{PDS} is in italics.

Elementary step (alternating)	Intermediate species	ΔG(TiN_4-G) /eV	ΔG(TiN_4B-G) /eV
* + N_2 = *N_2	*N_2	-0.41	-0.39
*N_2+(H^++e^-) =*NNH	*NNH	0.68	0.80
*NNH+(H^++e^-) =*NHNH	*NHNH	0.23	0.08
NHNH+(H^++e^-) =$NHNH_2$	*$NHNH_2$	-1.18	-1.18
$NHNH_2$+(H^++e^-) =NH_2NH_2	*NH_2NH_2	*1.24*	*1.01*
NH_2NH_2+(H^++e^-) =NH_2+NH_3	*NH_2	-2.8	-2.49
NH_2+(H^++e^-) =NH_3	*NH_3	0.77	0.54
* NH_3=*+NH_3	NH_3	0.69	0.85
**Elementary step (enzymatic)**	**Intermediate species**	**ΔG(TiN_4-G) /eV**	**ΔG(TiN_4B-G) /eV**
* + N_2 = *NN	*N_2	-0.50	-0.24
*NN+(H^++e^-) =*NNH	*NNH	0.37	0.34
*NNH+(H^++e^-) =*NHNH	*NHNH	-0.24	-0.16
NHNH +(H^++e^-) =$NHNH_2$	*$NHNH_2$	-0.3	-0.65
$NHNH_2$+(H^++e^-) =NH_2NH_2	*NH_2NH_2	-1.12	*1.04*
NH_2NH_2+(H^++e^-) =NH_2NH_3	*NH_2NH_3	-0.48	-2.58
NH_2NH_3+(H^++e^-) =NH_3+NH_3	*NH_3	*0.80*	*0.63*
* NH_3=*+NH_3	NH_3	0.69	0.85

Table S5. The Gibbs free energy for distal and alternating pathway of VN_4-G and VN_4B-G at U=0 V. The ΔG_{PDS} is in italics.

Elementary step (distal)	Intermediate species	ΔG(VN_4-G) /eV	ΔG(VN_4B-G) /eV
* + N_2 = *N_2	*N_2	-0.52	-0.44
*N_2+(H^++e^-) =*NNH	*NNH	*0.71*	*0.80*
NNH+(H^++e^-) =NNH_2	*NNH_2	-0.39	-0.4
*NNH_2+(H^++e^-) =*N+NH_3	*N	-0.25	-0.3
*N+(H^++e^-) =*NH	*NH	-1.16	-1.07
NH+(H^++e^-) =NH_2	*NH_2	-0.19	-0.41
NH_2+(H^++e^-) =NH_3	*NH_3	0.51	0.28
NH_3=+NH_3	NH_3	0.50	0.77
**Elementary step (alternating)**	**Intermediate species**	**ΔG(VN_4-G) /eV**	**ΔG(VN_4B-G) /eV**
* + N_2 = *N_2	*N_2	-0.52	-0.42
*N_2+(H^++e^-) =*NNH	*NNH	*0.71*	*0.82*
*NNH+(H^++e^-) =*NHNH	*NHNH	0.55	0.29
NHNH+(H^++e^-) =$NHNH_2$	*$NHNH_2$	-0.67	-0.51
$NHNH_2$+(H^++e^-) =NH_2NH_2	*NH_2NH_2	0.64	0.30
NH_2NH_2+(H^++e^-) =NH_2+NH_3	*NH_2	-2.50	-2.30
NH_2+(H^++e^-) =NH_3	*NH_3	0.51	0.29
* NH_3=*+NH_3	NH_3	0.50	0.76

Table S6. The Gibbs free energy for alternating and enzymatic pathway of CrN_4-G and CrN_4B-G at U=0 V. The ΔG_{PDS} is in italics.

Elementary step (alternating)	Intermediate species	ΔG(CrN_4-G) /eV	ΔG(CrN_4B-G) /eV
* + N_2 = *N_2	*N_2	-1.14	0.15
*N_2+(H^++e^-) =*NNH	*NNH	*0.65*	*0.88*
*NNH+(H^++e^-) =*NHNH	*NHNH	0.38	0.55
NHNH+(H^++e^-) =$NHNH_2$	*$NHNH_2$	-0.05	-0.59
$NHNH_2$+(H^++e^-) =NH_2NH_2	*NH_2NH_2	0.02	0.00
NH_2NH_2+(H^++e^-) =NH_2+NH_3	*NH_2	-1.80	-1.69
NH_2+(H^++e^-) =NH_3	*NH_3	-0.18	-0.32
* NH_3=*+NH_3	NH_3	1.34	0.24

Elementary step (enzymatic)	Intermediate species	ΔG(CrN_4-G) /eV
* + N_2 = *NN	*N_2	-0.67
*NN+(H^++e^-) =*NNH	*NNH	0.96
*NNH+(H^++e^-) =*NHNH	*NHNH	-0.23
NHNH +(H^++e^-) =$NHNH_2$	*$NHNH_2$	-0.57
$NHNH_2$+(H^++e^-) =NH_2NH_2	*NH_2NH_2	*1.08*
NH_2NH_2+(H^++e^-) =NH_2NH_3	*NH_2NH_3	-1.69
NH_2NH_3+(H^++e^-) =NH_3+NH_3	*NH_3	-0.99
* NH_3=*+NH_3	NH_3	1.34

Table S7. The Gibbs free energy for alternating and enzymatic pathway of ZrN_4-G and ZrN_4B-G at U=0 V. The ΔG_{PDS} is in italics.

Elementary step (alternating)	Intermediate species	ΔG(ZrN_4-G) /eV	ΔG(ZrN_4B-G) /eV
* + N_2 = *N_2	*N_2	-0.41	-0.33
*N_2+(H^++e^-) =*NNH	*NNH	0.69	0.68
*NNH+(H^++e^-) =*NHNH	*NHNH	0.00	0.02
NHNH+(H^++e^-) =$NHNH_2$	*$NHNH_2$	-1.40	-1.41
$NHNH_2$+(H^++e^-) =NH_2NH_2	*NH_2NH_2	*1.67*	*1.51*
NH_2NH_2+(H^++e^-) =NH_2+NH_3	*NH_2	-3.16	-2.96
NH_2+(H^++e^-) =NH_3	*NH_3	1.17	0.92
* NH_3=*+NH_3	NH_3	0.66	0.8
**Elementary step (enzymatic)**	**Intermediate species**	**ΔG(ZrN_4-G) /eV**	**ΔG(ZrN_4B-G) /eV**
* + N_2 = *NN	*N_2	-0.57	-0.44
*NN+(H^++e^-) =*NNH	*NNH	0.32	0.36
*NNH+(H^++e^-) =*NHNH	*NHNH	-0.34	-0.36
NHNH +(H^++e^-) =$NHNH_2$	*$NHNH_2$	-0.51	-0.59
$NHNH_2$+(H^++e^-) =NH_2NH_2	*NH_2NH_2	-1.43	*1.48*
NH_2NH_2+(H^++e^-) =NH_2NH_3	*NH_2NH_3	-0.22	-3.14
NH_2NH_3+(H^++e^-) =NH_3+NH_3	*NH_3	*1.33*	1.11
* NH_3=*+NH_3	NH_3	0.66	0.80

Table S8. The Gibbs free energy for alternating and enzymatic pathway of NbN_4-G and NbN_4B-G at U=0 V. The ΔG_{PDS} is in italics.

Elementary step (alternating)	Intermediate species	$\Delta G(NbN_4\text{-}G)$ /eV	$\Delta G(NbN_4B\text{-}G)$ /eV
$* + N_2 = *N_2$	$*N_2$	-0.62	-0.68
$*N_2+(H^++e^-) =*NNH$	*NNH	-0.17	0.12
$*NNH+(H^++e^-) =*NHNH$	*NHNH	-0.22	0.35
$*NHNH+(H^++e^-) =*NHNH_2$	$*NHNH_2$	-0.01	-0.84
$*NHNH_2+(H^++e^-) =*NH_2NH_2$	$*NH_2NH_2$	-1.76	-1.76
$*NH_2NH_2+(H^++e^-) =*NH_2+NH_3$	$*NH_2$	0.10	0.02
$*NH_2+(H^++e^-) =*NH_3$	$*NH_3$	*1.47*	*1.28*
$* NH_3=*+NH_3$	NH_3	0.44	0.73
**Elementary step (enzymatic)**	**Intermediate species**	**$\Delta G(NbN_4\text{-}G)$ /eV**	**$\Delta G(NbN_4B\text{-}G)$ /eV**
$* + N_2 = *NN$	$*N_2$	-0.46	-0.55
$*NN+(H^++e^-) =*NNH$	*NNH	0.38	0.52
$*NNH+(H^++e^-) =*NHNH$	*NHNH	0.44	0.33
$*NHNH +(H^++e^-) =*NHNH_2$	$*NHNH_2$	-1.40	-1.38
$*NHNH_2+(H^++e^-) =*NH_2NH_2$	$*NH_2NH_2$	*1.83*	*1.64*
$*NH_2NH_2+(H^++e^-) =*NH_2NH_3$	$*NH_2NH_3$	-3.35	-3.12
$*NH_2NH_3+(H^++e^-) =*NH_3+NH_3$	$*NH_3$	1.35	1.05
$* NH_3=*+NH_3$	NH_3	0.44	0.73

Table S9. The Gibbs free energy for alternating and enzymatic pathway of MoN_4-G and MoN_4B-G at U=0 V. The ΔG_{PDS} is in italics.

Elementary step (alternating)	Intermediate species	$\Delta G(MoN_4\text{-G})$ /eV	$\Delta G(MoN_4\text{B-G})$ /eV
* + N_2 = *N_2	*N_2	-0.58	-0.54
*N_2+(H^++e^-) =*NNH	*NNH	0.22	0.35
*NNH+(H^++e^-) =*NHNH	*NHNH	*0.95*	*0.63*
NHNH+(H^++e^-) =$NHNH_2$	*$NHNH_2$	-0.61	-0.41
$NHNH_2$+(H^++e^-) =NH_2NH_2	*NH_2NH_2	0.90	0.48
NH_2NH_2+(H^++e^-) =NH_2+NH_3	*NH_2	-2.76	-2.49
NH_2+(H^++e^-) =NH_3	*NH_3	0.86	0.53
* NH_3=*+NH_3	NH_3	0.25	0.67
**Elementary step (enzymatic)**	**Intermediate species**	**$\Delta G(MoN_4\text{-G})$ /eV**	**$\Delta G(MoN_4\text{B-G})$ /eV**
* + N_2 = *NN	*N_2	-0.68	-0.45
*NN+(H^++e^-) =*NNH	*NNH	0.17	-0.16
*NNH+(H^++e^-) =*NHNH	*NHNH	0.34	0.30
NHNH +(H^++e^-) =$NHNH_2$	*$NHNH_2$	-0.24	-0.13
$NHNH_2$+(H^++e^-) =NH_2NH_2	*NH_2NH_2	*1.21*	*1.08*
NH_2NH_2+(H^++e^-) =NH_2NH_3	*NH_2NH_3	-2.85	-2.90
NH_2NH_3+(H^++e^-) =NH_3+NH_3	*NH_3	1.04	0.84
* NH_3=*+NH_3	NH_3	0.25	0.67

Table S10. The Gibbs free energy for alternating pathway of RuN_4-G and RuN_4B-G at U=0 V. The ΔG_{PDS} is in italics.

Elementary step (alternating)	Intermediate species	ΔG(RuN_4-G) /eV	ΔG(RuN_4B-G) /eV
* + N_2 = *N_2	*N_2	-0.43	-0.38
*N_2+(H^++e^-) =*NNH	*NNH	*1.24*	*0.81*
*NNH+(H^++e^-) =*NHNH	*NHNH	-0.11	0.39
NHNH+(H^++e^-) =$NHNH_2$	*$NHNH_2$	0.28	-0.15
$NHNH_2$+(H^++e^-) =NH_2NH_2	*NH_2NH_2	-0.08	0.20
NH_2NH_2+(H^++e^-) =NH_2+NH_3	*NH_2	-1.52	-1.66
NH_2+(H^++e^-) =NH_3	*NH_3	-0.26	-0.27
* NH_3=*+NH_3	NH_3	0.10	0.28

Table S11. The Gibbs free energy for alternating and enzymatic pathway of HfN_4-G and HfN_4B-G at U=0 V. The ΔG_{PDS} is in italics.

Elementary step (alternating)	Intermediate species	$\Delta G(HfN_4\text{-}G)$ /eV	$\Delta G(HfN_4B\text{-}G)$ /eV
$* + N_2 = *N_2$	$*N_2$	-0.41	-0.41
$*N_2+(H^++e^-) =*NNH$	*NNH	0.49	0.55
$*NNH+(H^++e^-) =*NHNH$	*NHNH	0.00	-0.06
$*NHNH+(H^++e^-) =*NHNH_2$	$*NHNH_2$	-1.43	-1.44
$*NHNH_2+(H^++e^-) =*NH_2NH_2$	$*NH_2NH_2$	*1.99*	*1.83*
$*NH_2NH_2+(H^++e^-) =*NH_2+NH_3$	$*NH_2$	-3.55	-3.36
$*NH_2+(H^++e^-) =*NH_3$	$*NH_3$	1.53	*1.29*
$* NH_3=*+NH_3$	NH_3	0.60	0.82
**Elementary step (enzymatic)**	**Intermediate species**	**$\Delta G(HfN_4\text{-}G)$ /eV**	**$\Delta G(HfN_4B\text{-}G)$ /eV**
$* + N_2 = *NN$	$*N_2$	-0.58	-0.54
$*NN+(H^++e^-) =*NNH$	*NNH	0.19	0.22
$*NNH+(H^++e^-) =*NHNH$	*NHNH	-0.42	-0.41
$*NHNH +(H^++e^-) =*NHNH_2$	$*NHNH_2$	-0.53	-0.61
$*NHNH_2+(H^++e^-) =*NH_2NH_2$	$*NH_2NH_2$	-1.69	1.44
$*NH_2NH_2+(H^++e^-) =*NH_2NH_3$	$*NH_2NH_3$	-0.06	-3.24
$*NH_2NH_3+(H^++e^-) =*NH_3+NH_3$	$*NH_3$	*1.71*	*1.55*
$* NH_3=*+NH_3$	NH_3	0.60	0.82
